# Push-out bond strength of quartz fibre posts to root canal dentin using total-etch and self-adhesive resin cements

**DOI:** 10.4317/medoral.17429

**Published:** 2011-12-06

**Authors:** Mehdi A. Kahnamouei, Narmin Mohammadi, Elmira J. Navimipour, Maryam Shakerifar

**Affiliations:** 1 DDS, MSD, Assistant professor, Department of Operative Dentistry, School of Dentistry, Tabriz University of Medical Sciences, Tabriz, Iran; 2 DDS, MSD, Associate professor, Department of Operative Dentistry, School of Dentistry, Tabriz University of Medical Sciences, Tabriz, Iran; 3 DDS, MSD, Assistant professor, Department of Operative Dentistry, School of Dentistry, Tabriz University of Medical Sciences, Tabriz, Iran; 4 DDS, MSD, Assistant professor, Department of Operative Dentistry, School of Dentistry, Kerman University of Medical Sciences, Kerman, Iran

## Abstract

Objectives: Several adhesive systems are available for cementation of fibre posts into the root canal. The aim of the present study was to investigate the push-out bond strengths of quartz fibre posts to root dentin with the use of different total-etch and self-adhesive resin cements. Study Design: Ninety single-rooted human premolars were endodontically treated and standardized post-spaces were prepared. Fibre posts were cemented with different luting agents: total-etch (Nexus NX3, Duo-Link, and RelyX ARC) and self-adhesive resin cements (Maxcem Elite, BisCem, and RelyX Unicem). Three post/dentin sections (coronal, middle and apical) were obtained from each specimen, and push-out bond strength test was performed in each section at a cross-head speed of 0.5 mm/min. Data was analyzed with two-factor and one-way analysis of variance and a post-hoc Tukey test at a significance level of p < 0.05. Results: Cement type, canal region, and their interaction significantly influenced bond strength. Significantly higher bond strength values were observed in the apical region of self-adhesive cements. Only Duo-Link and RelyX ARC cements resulted in homogeneous bond strengths. Conclusions: Cementation of quartz fibre posts using self-adhesive cements provided higher push-out bond strengths especially in the apical region, while total-etch cements resulted in more uniform bond strengths in different regions of the root canal.

**Key words:** Push-out bond strength; quartz fibre post; total-etch resin cement; self-adhesive resin cement.

## Introduction

One of the most important problems in dentistry is the restoration of root-filled teeth. Posts and cores are frequently used in endodontically-treated teeth with excessive loss of coronal tooth structure. In such cases cementation of a post inside the root canal would provide retention for the final coronal restoration. Among the years different types of bonded posts, such as translucent fibre-reinforced posts, have been introduced. One of the main advantages is that the curing light can be transmitted through the post into the root dentin; however, significant differences in light transmission capability have been reported among fibre posts (1). These posts are capable of bonding to resin cements and transporting stresses that can be generated between the post and the root canal. Moreover, they have high fatigue and tensile strengths, and their stiffness (modulus of elasticity) is comparable to that of dentin and other fibre posts, resulting in fewer and more favorable root fractures (2,3). 

Three categories of luting agents are used for luting fibre posts into the root canal, mainly classified as total-etch, self-etch and self-adhesive resin cements. Total-etch resin cements require the separate use of phosphoric acid followed by multi- or two-step total-etch adhesives before the application of the resin cement (4). This system results in higher bond strengths on coronal dentin; although weak moisture control and incomplete resin inter-diffusion significantly decreases the bond strength to radicular dentin (5). Self-etch resin cements use an acidic primer which, without rinsing, can alter tooth structure before bonding; therefore, the clinical steps are simpler than those with total-etch cements (4). 

A new subgroup of resin cements, self-adhesive cements, was introduced in 2002. These materials were designed with the purpose of overcoming some of the limitations of both conventional and self-etch resin cements. Self-adhesive cements do not require any pretreatment of the tooth substrate: once the cement is mixed, application is accomplished in a single clinical step (6).

Limited investigations are available on the features of self-adhesive resin cements in comparison to the other multi-step luting agents.T Some studies were only limited to comparison of the bonding behaviour of different self-adhesive resin cements (4, 7-9). On the other hand, variations in the structure of root canal dentin, such as accessory root canals, areas of resorption, embedded and free pulp stones, and varying amounts of irregular secondary dentin, may affect bonding to different regions of the root (10). Therefore, the aim of the present study was to compare the push-out bond strength of quartz fibre posts to root dentin using three different brands of total-etch and self-adhesive resin cements in different regions of root canal dentin. 

## Material and Methods

Ninety single-rooted human premolars with mature apices, extracted for orthodontic reasons, were selected. The teeth were gathered following informed consent, approved by the Deputy Dean of Research at Tabriz Dental School. The roots of the teeth were examined under a stereomicroscope (Nikon, Model P-IBSS2 1002981, Japan) to rule out the presence of cracks and caries. The selected teeth had nearly the same dimensions, measured with digital calipers (Guanglu Measuring Instrument Co., China). Teeth with a coronal canal diameter more than the size of Gates Glidden #4 (MANI Inc., Tochigi, Japan) were excluded. The teeth were cleaned and stored in 0.5% chloramine T solution at 37ºC in an incubator for no longer than 1 month. Then the crowns of the teeth were removed 1 mm coronal to the cemento-enamel junction (CEJ) with a low-speed diamond saw under water spray.

The root canals were enlarged and prepared to a working length of 1 mm from the apical foramen by using k-files (MANI Inc.) and Gates Glidden burs #2 to #4 in a step-back technique. Irrigation was performed using 2.5% sodium hypochlorite solution and master apical file size was 35. The root canals were dried by using paper points (PT Dent, USA) and obturated with gutta-percha cones (PT Dent) and a eugenol-free sealer (AH-26; DeTrey, Zurich, Switzerland) using lateral condensation technique. Then the roots were fixed in a cylindrical mold of self-curing acrylic resin. The post spaces were prepared 24 hours after completing endodontic procedures. Gutta-percha was removed from the coronal part of the canals with Peeso reamers #2 and #3 (MANI Inc.) and subsequently, the post space was prepared up to 9 mm apical to the CEJ with a low-speed drill #3 provided by the manufacturer (RTD). The specimens were randomly divided into six groups of 15 roots, based on the luting agents used for the cementation of the posts. 

Quartz fibre posts (RTD ILLUSION #3; St. Egreve, France) were cleaned by using 70% ethanol and dried with air. Total-etch resin cements (Nexus NX3, Duo-Link, RelyX ARC) were used in three groups for cementation of fibre posts, and self-adhesive resin cements (Maxcem Elite, BisCem, RelyX Unicem) were used in three other groups. The procedures were carried out according to manufacturer’s instructions ( Table 1). It must be pointed out that a lentulo spiral (Dentsply Maillefer, Ballaigues, Switzerland) was used for insertion of the cements into the root canal. The posts were then seated to full depth in the prepared spaces using finger pressure and excess luting agent was removed with a small brush. Considering that all of the cements used in this study are dual-curing resins, the stable finger pressure was maintained for 60 sec to stabilize the fiber postes in the post spaces. After the initial chemical polymerization, the luting agent was

**Table 1:**
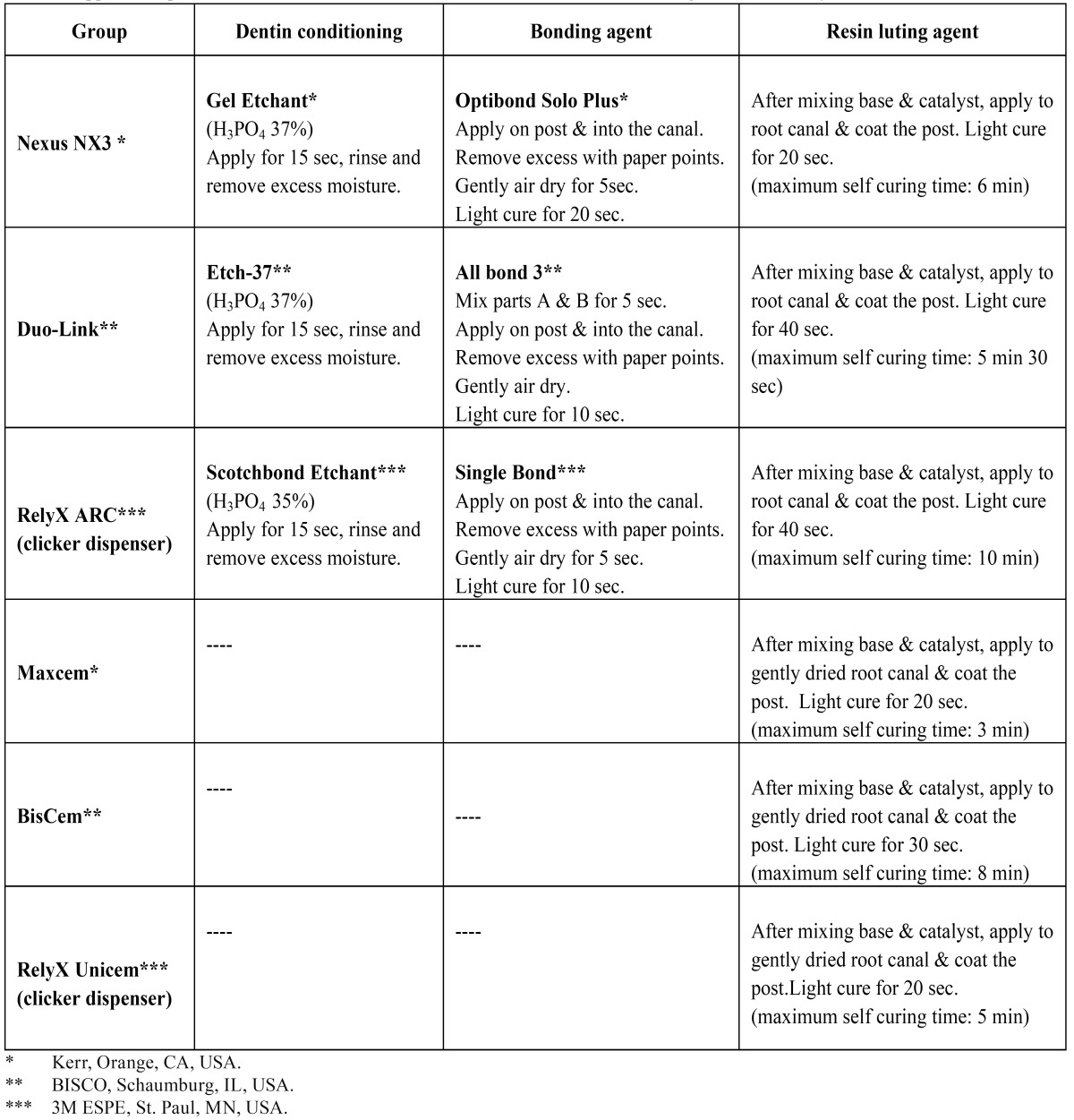
Application procedures of the total-etch and self-adhesive resin cements investigated in this study.

light polymerized through the posts, with the tip of the light unit in direct contact with the coronal ends of the posts. Light curing was performed using Astralis 7 (Ivoclar Vivadent AG, FL-9494 Schaan/Liechtenstein) at 700 mW/cm2 light intensity. The time of light curing in each group is mentioned in ( Table 1).

**Figure 1:**
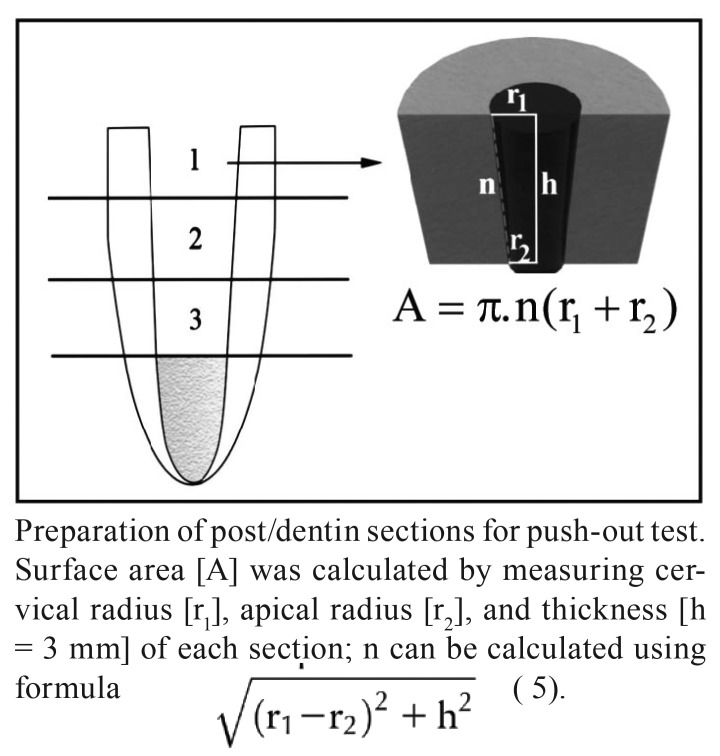
Total bonding area for each post segment.

All the specimens were stored in distilled water for 24 hours at 37ºC before testing. Then the specimens were sectioned using a low-speed saw (Isomet; Buehler Ltd, Lake Bluff, IL, USA) perpendicular to the long axis of the roots under water cooling. From each specimen, three 3-mm-thick post/dentin sections (cervical, middle, and apical) were obtained. Therefore a total of 45 specimens from each cement, consisting of 15 segments from each of the three different post space regions, were prepared. The exact thicknesses of fibre post segments were measured using a digital micrometer with 0.01-mm accuracy. Due to the tapered design of fibre posts, segments in each section had a truncated cone shape. For this reason, post diameters were measured on each surface of the post/dentin sections using digital calipers and the total bonding area for each post segment was calculated (Fig. 1). 

The sections were mounted in a universal testing machine (Hounsfield Test Equipment, H5K-S model; Surrey, England) with the apical surface upside and loaded until fracture at a cross-head speed of 0.5 mm/min. The punch was selected 0.2 mm smaller in diameter than the post as measured on the apical surface. The support of the section featured a cavity below the post, 0.2 mm wider than the post space. The peak force at the point of extrusion of the post segment from the test specimen was considered as the point of bond failure and recorded in Newton (N). Push-out bond strength values in MPa were then calculated by dividing the peak force by the bonded surface area (A) of the post segment.

Data was analyzed using two-factor analysis of variance to examine the main effects of the resin cement type, post space region, and the interaction between these two factors, and one-way analysis of variance to indicate the differences among the cements in each group. Pair-wise comparisons of the groups were performed using a post-hoc Tukey test (α = 0.05). 

## Results

The mean values of push-out bond strength values of the cements in different regions of the roots are shown in ( Table 2). Two-factor analysis of variance demonstrated that the bond strength was significantly influenced by the type of resin cement, the post space region and the interaction between these two factors (P<0.001). The mean value of push-out bond

**Table 2:**
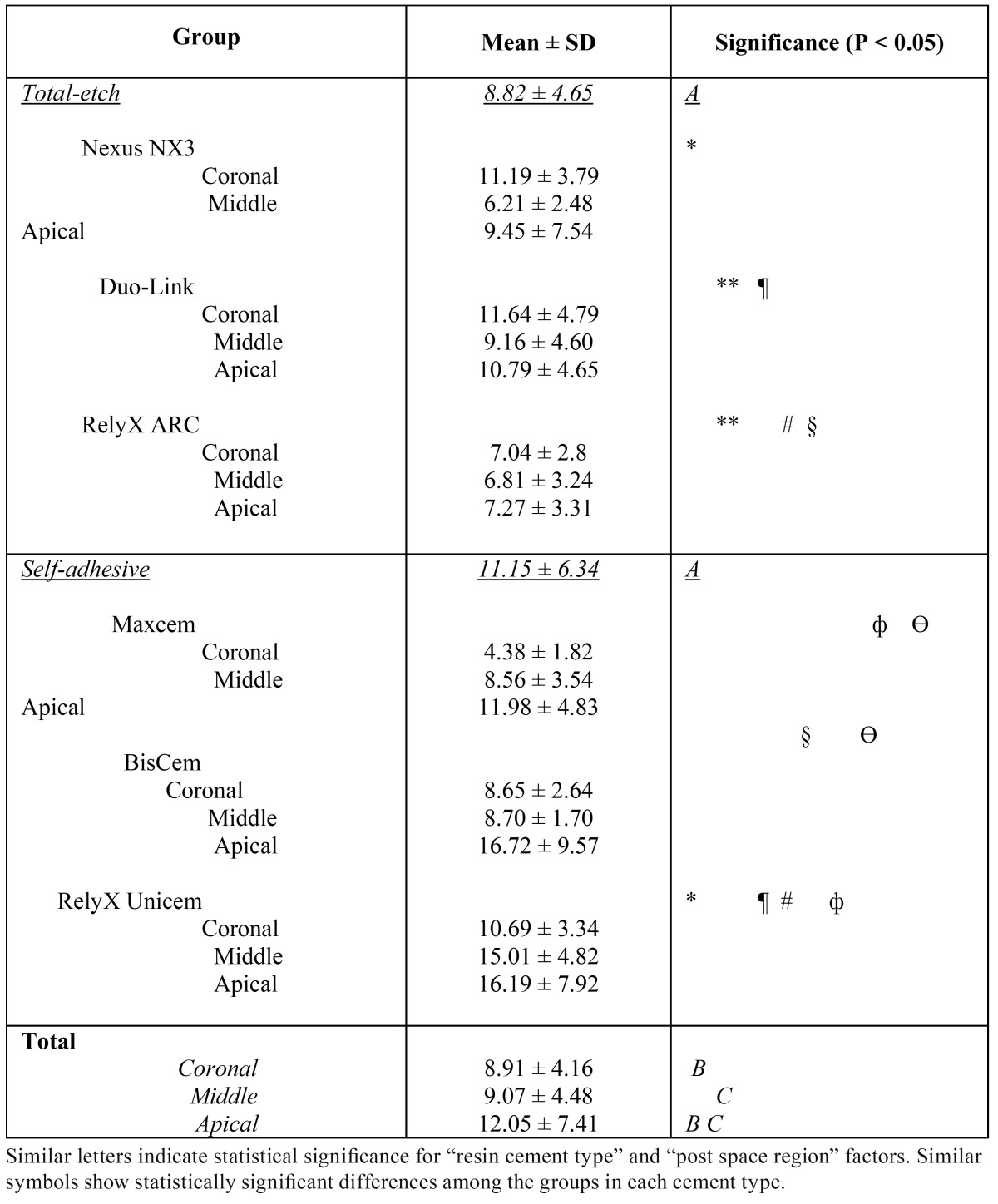
Push-out bond strength of total-etch and self-adhesive resin cements in different post space regions (in MPa).

strength was significantly higher in self-adhesive resin cements than total-etch ones (P< 0.001). Regarding the post space region, bond strength value in the apical third was significantly higher than those in the coronal or middle thirds (P<0.001), whereas there were no significant differences between the coronal and middle thirds (P=1). 

Comparison of the regions in each cement type showed that in total-etch resin cements there was a significant difference between coronal and middle third post space regions (P=0.02); furthermore, the same results were observed between the middle and apical post space regions (P=0.01). However, the difference between the coronal and apical areas was not significant (P=0.71). In self-adhesive resin cements, significant differences were observed between the coronal and middle, middle and apical, and coronal and apical post space regions (P<0.001). 

In comparison of each region between total-etch and self-adhesive resin cements, significant differences were observed in the coronal, middle or apical regions between both groups (P<0.001). In the coronal third a higher bond strength value was recorded in the total-etch group, whereas in the middle and apical regions it was higher in the self-adhesive group.

**Figure 2:**
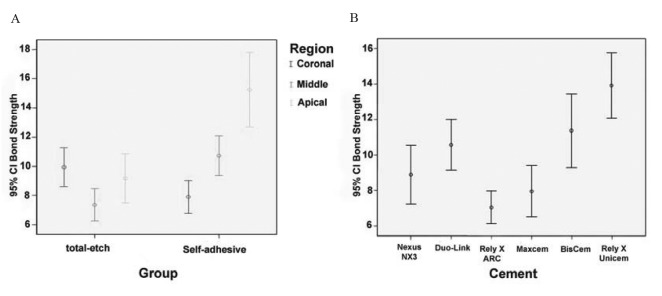
Error Bar charts of push-out bond strengths in: [A] different regions of the root according to resin cement types;[B] different resin cements under study.

Bond strength values were statistically different among all the cements under study (P<0.05). In ( Table 2) the significant differences in two-by-two comparisons are illustrated. The highest and the lowest push-out bond strength values were recorded with RelyX Unicem (13.91±5.99 MPa) and RelyX ARC (7.04±3.06 MPa), respectively. In other cements mean bond strength values were somewhere between these values (BisCem: 11.37±6.84 MPa, Duo-Link: 10.57±4.68 MPa, Nexus NX3: 8.89±5.34 MPa and Maxcem Elite: 7.95±4.56 MPa). (Fig. 2) separately shows the error bar charts of push-out bond strength values in different regions of the root canal in each cement type and in all the resin cements under study. 

## Discussion

Push-out tests produce a shear stress at the post-cement interface as well as at the dentin-cement interface. This is more comparable with the stresses under clinical conditions than the linear shear test (7). In addition, a study by Goracci et al. (8) showed that when measuring the bond strength of fibre posts adhesively luted to root canal dentin, the push-out test is more efficient and dependable than the microtensile technique. Therefore, the push-out design was used in the present investigation. 

According to the results of the present study, the bond strength was significantly higher in self-adhesive cements compared to total-etch systems and RelyX Unicem revealed the highest value among all the cements. Radovic

et al. (11) showed that RelyX Unicem is the most investigated self-adhesive cement in the published literature and its features are by far the most extensively explained by the manufacturer. Information about other brands is very limited. RelyX Unicem is mainly composed of glass fillers, silica, calcium hydroxide and methacrylated phosphoric esters. The adhesion mechanism is claimed to rely on both micromechanical retention and chemical interaction between monomer acidic groups and hydroxyapatite (11). The acid groups chelate the calcium ions of hydroxyapatite, promoting part of the chemical adhesion (12). Additionally, in order to assure neutralization of initial acidity of this cement a glass-monomer concept was applied, resulting in pH increase through reactions between phosphoric acid groups and alkaline fillers. Water formed during this process is claimed to contribute to the cement’s initial hydrophilicity, providing improved adaptation to dentin and moisture tolerance. Subsequently, water is reused by the reaction with acidic functional groups and during the cement reaction with ion-releasing basic fillers. Such a reaction would finally results in a hydrophobic matrix (11). Therefore, an ionic bond can also be formed between this cement and hydroxyapatite of the tooth that positively influences chemical bonding (13). These reasons might account for the good performance of self-adhesive cements, especially RelyX Unicem in the present study. In the present study BisCem showed the second highest bond strength after RelyX Unicem. According to manufacturer’s information it is mainly composed of Bis (hydroxyethyl metacrylate) phosphate and tetraethylene glycol dimethacrylate, which are hydrophilic components resulting in a better adaptation of cement to dentin (11). Higher bond strength values have been reported for the two above-mentioned cements among the other self-adhesive resin cements in other studies (11,14). In the present study specimens were stored in distilled water for 24 hours. In a study by Sadek et al. (15) significant increase in RelyX Unicem push-out strength was reported after 24 hours of water storage. On the other hand, moisture content after rinsing the root canal is difficult to control because of poor visibility. Furthermore, the narrow canal holds water by surface tension, making it difficult to replace water with bonding agents (16). Therefore, enhanced moisture content inside the root canal might have led to reduced bond strength values of total-etch systems, even though the root canals were dried carefully using paper points (7). 

Consistent with our results, a study carried out by Bitter et al. (7) showed that RelyX Unicem has a higher push-out bond strength value than either total-etch or self-etch resin cements. In another study, which compared the retention of quartz fibre posts using RelyX Unicem and RelyX ARC, the results showed that easy application and greater dependability of self-adhesive cements might improve bonding of fibre-based posts in root canals (17). 

In contrast to our results, Goracci et al. (8) showed that the push-out bond strength of Excite DSC/Variolink II to dentin after acid etching by phosphoric acid is higher than that of RelyX Unicem. They indicated that separate acid etching by using phosphoric acid is more effective at dissolving the thick smear layer of the root canal wall in comparison to the conditioning by weak methacrylated phosphoric esters in RelyX Unicem. However, it should be pointed out that in their study RelyX Unicem was self-cured whereas in the present study it was dual-cured. In another study by Wang et al. (9), better post retention was reported using total-etch cement (ONE-STEP PLUS/C&B CEMENT) compared to RelyX Unicem. Different study designs and various materials used might account for these discrepancies. Bonding to root canal dentin is affected by various factors, including dentin variations (18), mode of polymerization (19), compatibility between the resin cement and the bonding agent (20), irrigation solutions and sealers used for endodontic treatment (21), and the method of adhesive usage, e.g. size and kind of the microbrush (22).

Significant differences in bond strength values between total-etch and self-adhesive groups were observed in the present study, and significant differences even within the same category of luting cement were also noticed ( Table 2 and Fig. 2). It could be speculated that each cement possesses its own properties despite belonging to the same category of resin cements. In other studies, differences in bond strength of resin cements of the same category to radicular dentin have been reported (6,11,14). 

In the present study a higher bond strength value was achieved in the apical region, especially with self-adhesive resin cements. It seems that self-adhesive systems are less sensitive to dentin depth and tubular density than total-etch cements. Regarding tubule density in root dentin, Ferrari et al. (18) reported that tubule density is highest in the cervical region and significantly decreases in the middle and apical thirds. Some recent studies have reported that bond strength to root canal is not affected by the region in the root canal (8,23); however, further studies have revealed decreased bond strength values in the apical region (24,25). The results of the present study are consistent with the results of studies by Bitter et al. (7), Muniz and Mathias (21), and Gaston et al. (26), which have reported higher bond strength values in the apical third than those in other parts of the root canal. Therefore, in self-adhesive cements bond strengths to root dentin seem to be related more to the area of solid dentin than to the density of dentinal tubules (7,23,26). Conversely, in the present study bond strength to the coronal region was higher in total-etch systems than self-adhesive ones. It is likely that phosphoric acid etching is more effective in highly tubular areas of the coronal root dentin because it removes the thick surfaces smear layer and the smear plug in dentinal tubules formed during post space preparation to allow more effective micromechanical retention of resin cements (27-29). Similarly, a study by Perdigao et al. (30) showed higher bond strength values in the coronal region when using total-etch cements. Difficulties in conveying sufficient amount of primer-adhesive solution to the apical region of canals and manipulation problems arising from inadequate root canal access are the reasons for lower apical bond strength values in total-etch cements compared to self-adhesive ones (25,28,31,32). 

In the present study only dual-curing resin cements were evaluated. This way, the differences in polymerization method were controlled since it has been established that polymerization shrinkage influences bond strength. Flowability of the adhesive agent is one way to reduce polymerization shrinkage, which is dependent on the configuration of the preparation or C-factor. The C-factor (ratio of bonded to non-bonded surface) associated with the posts may exceed 200 (25). Therefore, in such situations resin cements with different polymerization methods may induce different values of stresses in bonding interface.

Finally, it should be pointed out that all the in-vitro studies have limitations and cannot completely replace clinical trials. More in-vitro studies are required to evaluate the durability of the bond after aging, and long-term clinical studies are needed before any clinical recommendations.

Within the limitations of this laboratory investigation, it can be concluded that cementation of quartz fibre posts with self-adhesive cements provides higher push-out bond strengths, especially in the apical region, while, total-etch cements result in more uniform bond strengths in different regions of the root canal.
